# Gut microbiota diversity is altered in a sex-dependent manner in Shank3B heterozygote mice

**DOI:** 10.3389/frmbi.2025.1628819

**Published:** 2025-06-30

**Authors:** Finley Turner, Mykle Williams, Sophie Gregoretti, Delano Bielamowicz, Kylie Roach, Lil Gehner, Anjali Kunnatha, Shekinah Phillips, Rosie Hagel, Rebecca Harshman, Erika Vargo, Stacey B. B. Dutton, Jennifer Kovacs, Jennifer Larimore

**Affiliations:** Department of Neuroscience and Philosophy, Agnes Scott College, Decatur, GA, United States

**Keywords:** microbiome, schizophrenia, autism spectrum disorder, gut-brain axis, neurodevelopmental disorders

## Abstract

The gut-brain axis is a dynamic interface that has been implicated in the pathogenesis and severity of various neurodevelopmental disorders such as schizophrenia (SZ) and autism spectrum disorders (ASD). Also implicated in ASD and SZ, *SHANK3B* is a critical gene for postsynaptic protein scaffolding at excitatory synapses. Shank3B knockout mice not only exhibit ASD-like behaviors but demonstrate altered gastrointestinal epithelium morphology and fecal microbiota composition. Utilizing Shank3B heterozygote mice to better reflect the clinical presentation of ASD, we sequenced the gut microbiome from the small intestine of 12-week-old wild type Shank3B^+/+^ or Shank3B^+/-^ mice in a sex-dependent manner, analyzing bacterial phyla, classes, orders, families, genera, and species. *Firmicutes* emerged as the dominant phylum in Shank3B^+/-^ mice and *Bacilli* as the dominant class, with *Lactobacillales* as the dominant order. The dominant family is *Lactobacillaceae*. The Shank3B^+/-^ males but not the Shank3B^+/-^ females show an increase in *Staphylococcaceae* and *Erysipelotricaceae*. Our results indicate increased biodiversity in Shank3B^+/-^ males and reduced biodiversity in Shank3B^+/-^ females compared to wild-type controls. Altogether, this data reveals sex-specific microbial signatures that may contribute to the pathogenesis of ASD thus providing potential therapeutics that target gut microbiota in neurodevelopmental disorders.

## Introduction

The gut-brain axis is an integral pathway contributing to nervous system development, behavioral regulation, and is important for understanding the pathogenesis of neurological disease (Ashique et al., 2024; Vuong and Hsiao, 2017; Kelly et al., 2021). Gut bacteria synthesize neurotransmitters such as serotonin (5-HT) (Wang and Wang, 2016). Additionally, certain gut bacteria, including *Corynebacterium glutamicum*, *Brevibacterium lactofermentum*, and *Brevibacterium avium*, possess glutamate racemase, enabling the conversion of L-glutamate to D-glutamate (Averina et al., 2020; Chang et al., 2020; Dicks, 2022). Research involving Alzheimer’s disease and Parkinson’s disease patients suggests that D-glutamate metabolized by gut bacteria may influence glutamate N-methyl-D-aspartate receptor (NMDAR) function and cognitive abilities (Chang et al., 2020). Furthermore, recent research focused on dysbiosis highlights the bidirectional relationship of the gut-brain axis (Rhee et al., 2009). Dysbiosis increases neuroinflammation and microglial activation (Ashique et al., 2024) and can result in hippocampal myelin damage (Tang et al., 2024). These studies underscore the importance of understanding the impact of altered gut microbiota on the nervous system.

Mouse models have been pivotal in elucidating the relationship between the gut microbiome and brain function. Mice with abnormal levels of the metabolite 4-ethylphenyl sulfate (4EPS), produced by the gut microbiome from tyrosine, exhibit altered oligodendrocyte activity, reduced myelination, and anxiety-like behavior (Needham et al., 2022). Germ-free mice showed altered social preferences and increased exploratory behavior but heightened stress responses. Remarkably, colonization with a typical microbiome partially reversed these behavioral abnormalities (Vuong et al., 2017). Furthermore, researchers demonstrate that mice colonized with microbiota from chronically stressed mice exhibited anxiety-like and depression-like behaviors and displayed reduced *Lactobacillus* and increased pro-inflammatory *Akkermansia* (Li et al., 2019).

Understanding the contributions of gut microbiota to the symptomatology and comorbidity of schizophrenia (SZ) and ASD presents promising avenues for novel therapeutic development. Both SZ and ASD are associated with learning deficits, increased anxiety, and developmental delay. Mouse models for these diseases demonstrate these phenotypes as well as alterations in the microbiome. Mice colonized with gut microbiota from individuals with autism spectrum disorder (ASD) exhibited ASD-like behaviors (Sharon et al., 2018). While the causal relationship between gut microbiome alterations and ASD pathology remains unknown, shared microbiome signatures, similar immune markers, and increased gut permeability have been observed in ASD, attention-deficit/hyperactivity disorder (ADHD), and comorbid ASD-ADHD cases (Di Gesù and Buffington, 2024). Notably, decreased levels of *Coprobacter* and *Howardella* and increased abundance of *Eggerthella*, *Hungatella*, and *Ruminococcus gnavus* suggest microbiome-mediated mechanisms may contribute to the overlapping features of these disorders (Di Gesù and Buffington, 2024). Alterations in gut microbiota composition have been consistently correlated with symptoms of both ASD and SZ (Cocean and Vodnar, 2024; Lin et al., 2024). Children with ASD show reduced levels of the bacterial genus *Bifidobacterium*, particularly *B. longum* (Duque et al., 2021). Studies in both humans with ASD and a mouse model of ASD report significant changes in genera such as *Akkermansia*, *Bacteroides*, *Bifidobacterium*, *Blautia*, *Clostridium*, *Dorea*, *Parabacteroides*, *Prevotella*, and *Lactobacillus* (Alamoudi et al., 2022). Various stressors and host factors such as diet, pharmaceuticals, toxins, pathogens, immune responses, and physical and psychological conditions are known to decrease microbial diversity and promote the growth of pathogenic taxa, leading to gut dysbiosis (Di Gesù and Buffington, 2024). This imbalance in gut microbial communities results in increased intestinal permeability and inflammation, contributing to neurological impairments, autoimmune responses, and gastrointestinal dysfunction commonly observed in individuals with ASD and SZ (Di Gesù and Buffington, 2024; Ansari et al., 2024; Lin et al., 2024).

The Shank family of proteins, particularly Shank3B, is integral to synapse formation and synaptic plasticity at glutamatergic synapses. *SHANK3* disruption is implicated in Phelan-McDermid Syndrome (23q13 deletion syndrome), non-syndromic ASD (Peça et al., 2011), and SZ (Rees et al., 2012; Gauthier et al., 2010). *SHANK3B* encodes key postsynaptic density (PSD) proteins crucial for glutamatergic synapses, linking ionotropic NMDA receptors to metabotropic mGlu5 receptors—a connection vital for synaptic plasticity (Jiang and Ehlers, 2013; Mullin et al., 2013; Grabrucker et al., 2011; Foss-Feig et al., 2017; Heavner et al., 2021). Reduced Shank3B protein levels result in spatial memory deficits and altered excitatory neurotransmission (Balaan et al., 2019; Brown et al., 2018; Cope et al., 2023; Dhamne et al., 2017). Shank3B knockout mice exhibit ASD-like behaviors, including compulsive grooming, abnormal social interactions, anxiety-like behaviors, and auditory processing deficits, paralleling traits observed in ASD (Balaan et al., 2019; Dhamne et al., 2017; Rendall et al., 2019). Significantly altered gastrointestinal (GI) epithelium morphology and fecal microbiota composition in Shank3 knockout mice has been reported, with an increase in *Actinobacteria* and *Firmicutes* and decreased *Proteobacteria* and *Verrucomicrobia*. Additionally, Shank3αβ knockout mice uniquely harbored *Deferribacteres*, *Tenericutes*, and *Chlamydiae* (Sauer et al., 2019).

Given this context, we set out to take the Sauer et al. work in the knockout mice further using the heterozygote mice instead and exploring the role of sex. We hypothesize that Shank3B+/- mice exhibit sex-specific gut microbiome alterations that may contribute to the pathophysiology of neurodevelopmental disorders. While knockout models provide valuable and necessary mechanistic insights, heterozygous models more accurately reflect the clinical presentation of neurodevelopmental disorders such as ASD and SZ, making them necessary for translational research.

## Materials and methods

### Mice

Mice were handled according to the approved IACUC protocol for this study. Mice were purchased from Jackson Labs (Bar Harbor, Maine, Strain #:017688, B6.129-Shank3tm2Gfng/J) and housed at Agnes Scott College according to standard protocols. Genotype PCR confirmed the genetics identified by Jackson labs at the time of shipment. All mice used were littermates to control for cage effects. Females were tested to ensure that they were in the same phase of estrous at the time of sample collection. For genotyping, the primers used were referenced on Jackson Lab’s website. The common primer was GAGACTGATCAGCGCAGTTG, the wild type primer was TGACATAATCGCTGGCAAAG, and the mutant primer was GCTATACGAAGTTATGTCGACTAGG.

### Sample collection

At 12 weeks postnatal, animals were anesthetized using CO_2_ and rapidly decapitated. Small intestines were dissected and transferred to ice cold phosphate buffered saline (PBS) with proteinase K (Sigma Aldrich). Samples were stored at -80°C before shipment. In total, 3 wild type male mice (Shank3B^+/+^ C57B/6), 3 wild type female mice (Shank3B^+/+^ C57B/6), 3 heterozygote male mice (Shank3B^+/-^), and 3 heterozygote female mice (Shank3B^+/-^) were utilized. DNA was extracted and amplified using 16S and ITS2 rDNA bacterial tag-encoded FLX amplicon pyrosequencing (bTEFAP) using an Illumina MiSeq 20,000 sequence diversity assay (MRDNA, Shallowater, TX). The 16S rRNA gene V4 variable region PCR primers 515/806 and ITS2 with the barcode on the forward primer. Sequencing was performed at MR DNA (www.mrdnalab.com, Shallowater, TX, USA) on a MiSeq following the manufacturer’s guidelines. Sequence data were processed using DADA2/QIIME2. DADA2 denoising in QIIME2 was performed using the following parameters: forward and reverse reads were each trimmed by 20 bases at the 5’ end (–p-trim-left-f 20, –p-trim-left-r 20) and truncated to 200 bases (–p-trunc-len-f 200, –p-trunc-len-r 200) based on quality profiles. All other parameters, including chimera removal, were set to QIIME2 defaults. Briefly, QIIME2’s default settings for DADA2 denoising include chimera removal using the consensus method, a minimum required overlap of 12 nucleotides between forward and reverse reads after truncation, and error model learning based on up to 1,000,000 reads sampled from the dataset. For our sequences, no bases are trimmed from the start of reads (–p-trim-left-f 0, –p-trim-left-r 0), no quality-based truncation is applied (–p-trunc-q 2), and the maximum expected errors per read was set to 2 (–p-max-ee 2). The taxonomic identification was performed using the Greengenes2 2022.10 full-length database with a Naive Bayes classifier. We used the R package DESeq2 to perform pairwise comparisons between sample groups (Love et al., 2014) and the R package pheatmap to generate the heat map (pheatmap: Pretty Heatmaps. R package version 1.0.12, https://github.com/raivokolde/pheatmap). In summary, raw sequence data were demultiplexed, quality filtered, and denoised using QIIME2, with paired-end reads truncated and trimmed before amplicon sequence variant (ASV) inference via DADA2. Taxonomic classification was performed using a pre-trained Greengenes classifier, followed by filtering of non-target sequences, diversity analyses, and export of processed data for downstream analysis in R (Bolyen et al., 2019; Callahan et al., 2016).

### Statistical analyses

Alpha diversity was analyzed using the Shannon diversity index and observed richness. Analysis of variance (ANOVA) was conducted to determine significant shifts in alpha diversity across treatments and generations. Beta diversity was assessed using Bray-Curtis dissimilarity matrices with Principal Coordinates Analysis (PCoA) used for visualization. Permutational Multivariate Analysis of Variance (PERMANOVA) was performed to statistically evaluate the effects of treatment and generation on microbial community composition. Differential abundance testing was carried out using ANCOMBC to identify specific taxa that were significantly enriched or depleted under different conditions. Pairwise Wilcoxon tests with Benjamini-Hochberg (BH) correction were used for *post-hoc* comparisons of alpha diversity.

## Results

The gut-brain axis underscores the complex interactions between the CNS and the enteric nervous system, potentially contributing to the pathogenesis of various neurological disorders. Sauer et al. reported significantly altered gastrointestinal (GI) epithelium morphology and fecal microbiota composition in Shank3 knockout (KO) mice, with increased *Actinobacteria* and *Firmicutes* and decreased *Proteobacteria* and *Verrucomicrobia* (Sauer et al., 2019). Knockout model systems are necessary for mechanistic understanding, but heterozygous models reflect the clinical presentation of ASD. To explore the gut-brain axis further, we examined the gut microbiome of Shank3B^+/-^ mice in a sex-dependent manner.

**Figure 1:**
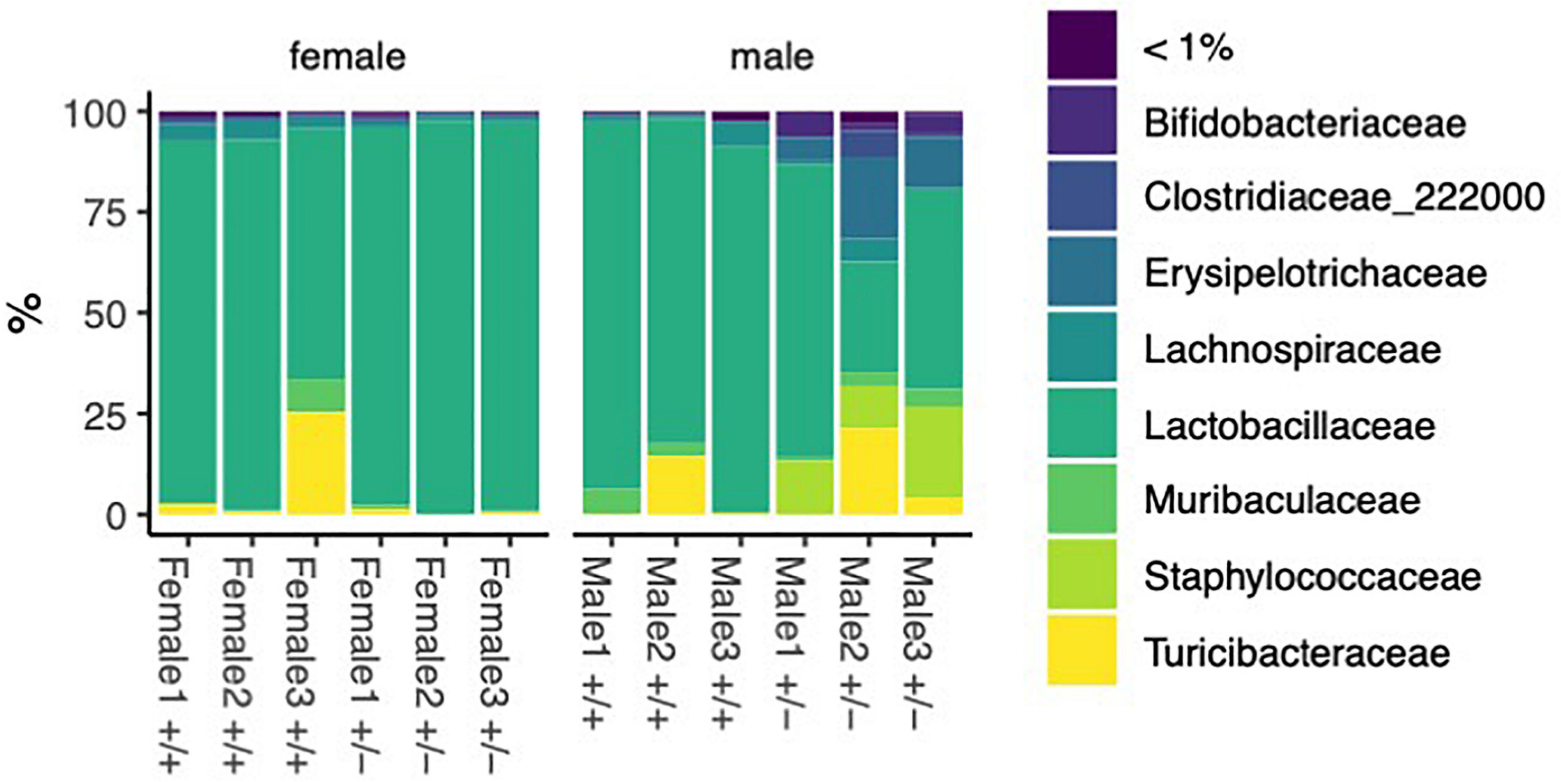
Bacteria identified in female and male Shank3B^+/+^ and Shank3B^+/-^ mice. Percentage bar graph of different bacterial families sequenced from the small intestine 3 wild type male mice (Shank3B^+/+^), 3 wild type female mice (Shank3B^+/+^), 3 heterozygote małe mice (Shank3B^+/-^), and 3 heterozygote female mice (Shank3B^+/-^).

To do this, we describe the phylum, class, order, family, genus, and species of gut microbiota isolated from the small intestines in 12-week-old Shank3B^+/+^ or Shank3B^+/-^ mice in a sex-dependent manner using whole-bacterial population sequencing (**Figure 1** and **Supplementary Figure 1**). **Supplementary Figure 1A** is a percentage bar graph of the bacterial phylum present in the small intestine samples. We see a clearly dominant *Firmicutes* phylum. These results are similar to the observations in the knockout mice which reported an increase in *Firmicutes* in the Shank3 KO (Sauer et al., 2019). The bacterial classes (**Supplementary Figure 1B**) found in the small intestines were analyzed, with the dominant class for all groups being *Bacilli*. The dominant bacterial order in the samples tested is *Lactobacillale*s (**Supplementary Figure 1C**). The Shank3B^+/-^ males show an increase in *Staphylococcales*, *Haloplasmatales*, and *Erysipelotricales*. The dominant family (**Figure 1**) is *Lactobacillaceae* and the Shank3B^+/-^ males show an increase in *Staphylococcaceae* and *Erysipelotricaceae*. This increase is not observed in the Shank3B^+/-^ female mice. Finally, we analyzed the genus and species of the bacteria sequenced from the small intestine of our samples (**Supplementary Figures 1D, E**). Present in the samples are *Bifidobacteriaceae*, *Staphylococcacae*, and *Turicibacteracea*. We observe an increase of these bacteria in the male Shank3B^+/-^ mice compared to control mice, but a decrease of these bacteria in the female Shank3B^+/-^ mice compared to control mice. Previous studies in both humans and mice show alterations in abundance for several genera including *Akkermansia, Bacteroides, Bifidobacterium, Parabacteroides* and *Prevotella* (Alamoudi et al., 2022).

**Figure 2:**
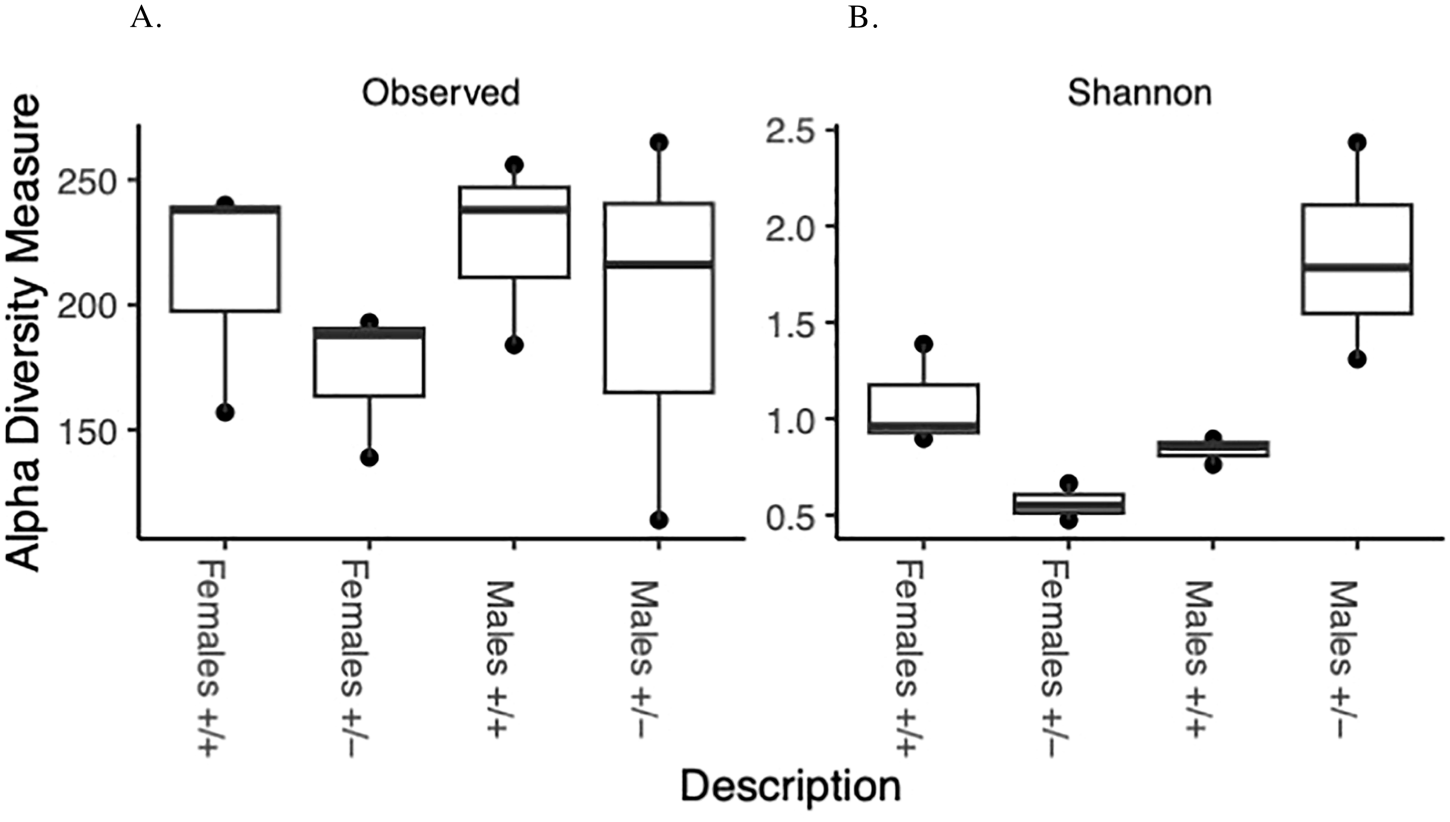
Alpha diversity: Box and whisker plot depicting overall species richness and shannon diversity index in male and female Shank3B^+/+^ and Shank3B^+/-^ mice. This comparison indicates how the relative diversities of each group compare to the diversity of the overall sample indicated by the **(A)** Species Richness and **(B)** Shannon diversity index.

The Alpha diversity measures show different patterns between the genotypes and sexes. The Shannon diversity exhibits significant differences between genotypes and sexes (**Figure 2**). Shannon diversity was analyzed with a two-way ANOVA. There is a significant main effect of sex on Shannon diversity (p = 0.0224). There is a highly significant interaction between genotype (treatment) and sex (p = 0.0032). The genotype (treatment) alone does not have a significant main effect (p = 0.2222). Observed richness does not show clear differences between groups (**Figure 2**). Neither genotype (treatment), sex, nor their interaction showed significant effects on observed richness (all p > 0.05). The discrepancy between Shannon’s diversity and observed richness can be explained by the following:

1. Sensitivity to rare species: Shannon’s index is more sensitive to rare species, while observed richness only counts the number of species present.
2. Evenness consideration: Shannon’s index accounts for both richness and evenness, whereas observed richness only considers the number of species.
3. Abundance information: Shannon’s index incorporates relative abundance information, while observed richness does not.
4. Resolution: Shannon’s index may detect subtle changes in community structure that are not reflected in simple species counts.

**Figure 3:**
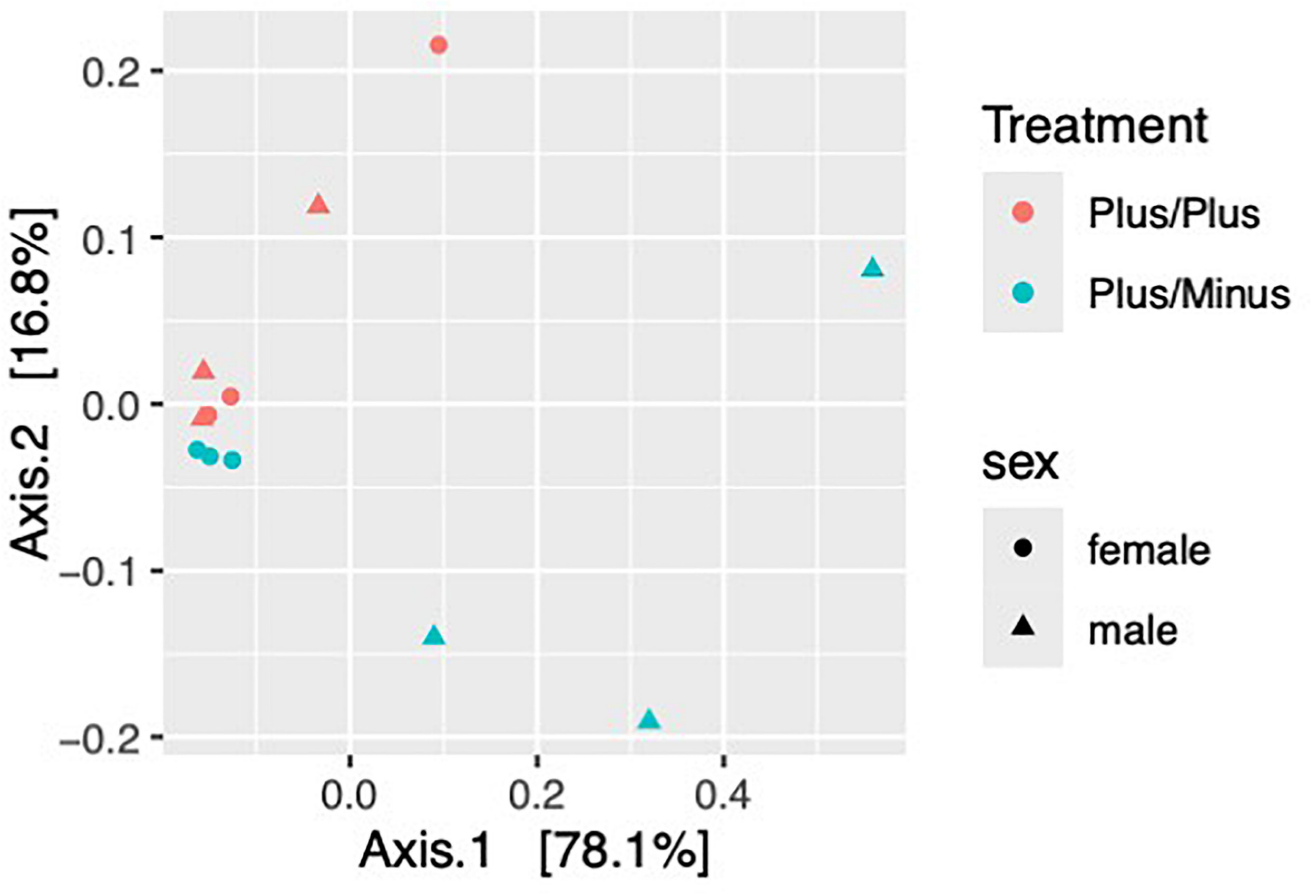
Beta diversity of samples from male and female Shank3B^+/+^ and Shank3B^+/-^ mice. Bray-Curtis Dissimilarity– Beta DiversityPCoA demonstrates that Shank3B^+/+^ females, Shank3B^+/+^ males, and Shank3B^+/-^ females are more similar in their overall microbiome composition then they are to Shank3B**^+/-^** males.

The treatment in this study compares two different genotypes, which show significant effects on the microbial community composition. The Beta diversity analysis (PERMANOVA, **Figure 3**) using Bray-Curtis dissimilarity shows a marginally significant effect of genotype (R2 = 0.40792, p = 0.058), no significant effect of sex (R2 = 0.00292, p = 0.941) and a significant interaction between genotype (treatment) and sex (R2 = 0.64994, p = 0.003).

The PERMANOVA results reveal important insights into how genotype and sex influence the microbial community composition in this study. First, the genotype (treatment) shows a marginally significant effect on microbial community composition with an R2 = 0.40792, indicating that genotype explains about 40.8% of the variation in microbial communities and a P-value = 0.058, which is close to the conventional significance threshold of 0.05. This suggests that different genotypes tend to harbor distinct microbial communities, and the genetic background of the host plays a substantial role in shaping the microbiome. Further investigation with a larger sample size might reveal more definitive genotype-specific patterns. Second, sex alone does not significantly influence microbial community composition with an R2 = 0.00292, explaining only 0.3% of the variation and a P-value = 0.941. This indicates that male and female mice, when considered independently of genotype, do not harbor significantly different microbial communities. Sex-specific factors may not be as influential as genetic factors in shaping the microbiome in this study. Finally, the interaction between genotype and sex shows a significant effect with an R2 = 0.64994, explaining 65% of the variation and a highly significant P-value = 0.003. This interaction suggests that the effect of genotype on microbial communities differs between males and females. Sex-specific responses to genetic variations may be crucial in determining microbiome composition.

**Figure 4:**
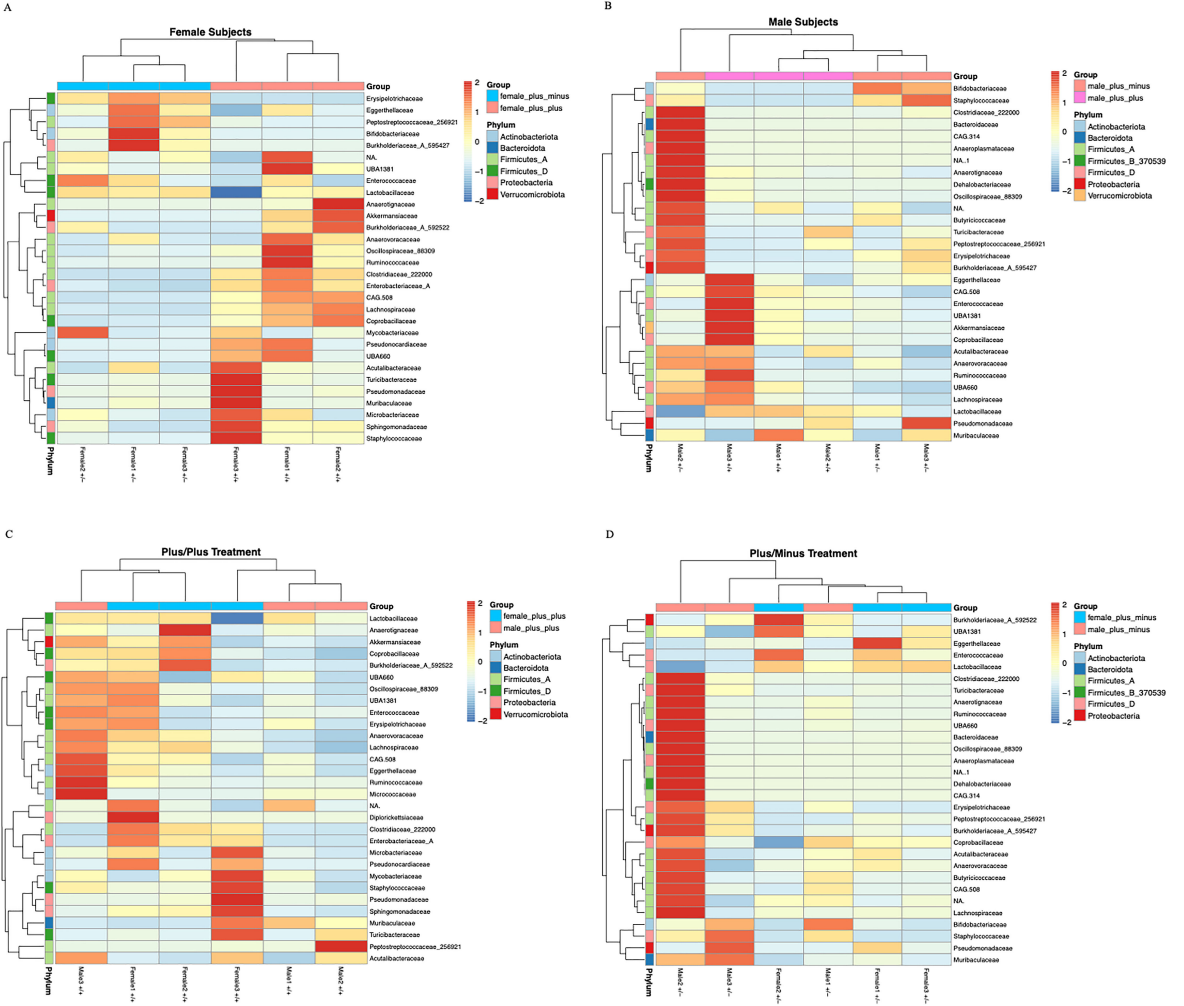
Heatmaps of the top 30 differentially abundant bacterial families across **(A)** female Shank3B^+/+^ and Shank3B^+/-^ mice **(B)** male Shank3B^+/+^ and Shank3B^+/-^ mice **(C)** all Shank3B^+/+^ mice and **(D)** all Shank3B^+/-^ mice. Each heatmap displays the relative abundance (row Z-scores) of the 30 most signicant bacterial families, as identied by DESeq2 differential abundance analysis (adjusted p-value < 0.05), across samples grouped by sex and treatment. Rows represent bacterial families, colored by their phylum annotation. Columns represent individual samples.

Finally, the heatmaps contrast the top 30 most significant bacterial families between treatments within each sex (**Figures 4A, B**), as well as between the sexes within treatment (**Figures 4C, D**). Overall, they reveal the significant interactions between sex and treatment on microbiome composition. While highlighting the overall increase in microbial taxa in male Shank3B^+/-^ mice and overall decrease in microbial taxa in female Shank3B^+/-^ mice relative to Shank3B^+/+^ males and females.

These differences suggest that the genotype treatments may be affecting the evenness or relative abundances of microbial species rather than just their presence or absence. The significant interaction between genotype and sex in the Beta diversity analysis further supports the complex nature of these effects on the microbial community structure.

## Discussion

Gastrointestinal disturbances are prevalent in neurodevelopmental disorders, including SZ and ASD (Severance et al., 2015; Alamoudi et al., 2022; Ansari et al., 2024; Zajkowska et al., 2024). Previous studies in both humans with ASD and mouse models of ASD demonstrate alterations in abundance for several genera including *Akkermansia, Bacteroides, Bifidobacterium, Parabacteroides* and *Prevotella* (Alamoudi et al., 2022). In this study, we analyzed the phyla, class, order, family, genus and species of bacterial sequenced from small intestine samples of Shank3B^+/+^ or Shank3B^+/-^ mice in a sex-dependent manner. We observed a sex-dependent difference, with increased biodiversity in the males compared to the females who demonstrated a decrease in biodiversity. Specifically, we observed an increase of *Bifidobacteriaceae*, *Staphylococcacae*, and *Turicibacteracea* in the male Shank3B^+/-^ mice compared to control mice, but a decrease of these bacteria in the female Shank3B^+/-^ mice compared to control mice. The interaction between genotype and sex shows a significant effect (R2 = 0.64994 and P-value = 0.003). This interaction suggests that the effect of genotype on microbial communities differs between males and females due to hormonal levels. Sex-specific responses to genetic variations may be crucial in determining microbiome composition.

16s rRNA sequencing, while widely used for gut microbiome analysis and characterization across a wide variety of taxa does have several limitations which can impact the taxonomic resolution of the microbes being characterized and the functional insights about those microbes. For example, short-read sequencing of hyper variable regions, like 16s, may be reliable for species level identification as it may not be able to distinguish between closely related species (Bailén et al., 2020). Additionally, our microbial identification pipeline is database dependent, and is therefore unable to identify microbes that lack reference sequences resulting in unclassified ASVs. This is particularly true for uncultured or novel species (Winand et al., 2019). These limitations should be considered in future studies.

Understanding the impact of sex and genetics on microbial communities is crucial for our understanding of neurodevelopment. The severity of SZ symptoms has been correlated with alterations in gut bacterial taxa, specifically reduced levels of *Veillonellaceae* and *Lachnospiraceae* (Zheng et al., 2019). Notably, SZ patients who received co-supplementation with vitamin D and probiotics exhibited significant improvements on the Positive and Negative Syndrome Scale (Ghaderi et al., 2019; Cocean and Vodnar, 2024). Children aged 5–9 years with ASD experienced reduced severity of symptoms on the Autism Treatment Evaluation Checklist after three months of consuming a probiotic formula (Cocean and Vodnar, 2024). Furthermore, six months of probiotic treatment led to enhancements in brain wave activity associated with working memory, analytical thinking, and sensory processing (Cocean and Vodnar, 2024). Another study analyzed probiotics in children with ASD aged 2 to 10 years. The ASD treated group demonstrated a positive correlation was observed between ABC total scores and Gastrointestinal Symptom Rating Scale (GSRS) scores, indicating that more severe autistic behaviors were associated with increased gastrointestinal symptoms (Lin et al., 2024). Taken together, these findings suggest that alleviating gastrointestinal symptoms may help reduce core ASD behaviors.

Future experiments include examining how the gut microbiome impacts behavioral phenotypes in a sex-dependent manner. Previous research has demonstrated that knockout of Shank3B results in spatial memory deficits, increased anxiety, and alterations in excitatory neurotransmission (Balaan et al., 2019; Brown et al., 2018; Cope et al., 2023; Dhamne et al., 2017). Previous work in the open field test (OFT) demonstrated Shank3B^−/−^ mice showed similar levels of activity and thigmotaxis compared to control, but, a significant reduction in rearing, a form of vertical exploration considered to be anxiogenic for mice (Peça et al, 2011). This work was done in the knockout mouse, so additional work in Shank3B^+/-^ mice and in a sex-dependent manner is necessary.

A better understanding of gut morphology is also necessary. It has been reported that Shank3B^−/−^ mice exhibit significantly altered epithelial morphology and increased GI permeability and compared to Shank3B^−/−^ mice, Shank3B^+/−^ mice exhibit mild epithelial alterations (Eberly et al., 2025). This report is one of the first to analyze the Shank3B^+/−^ mice, however, sex differences were not analyzed. Future studies examining sex-dependent differences of small intestine morphology are necessary to better understand the impact of these findings.

Future experiments also include examining the metabolite profile and microbiome profile during key developmental stages. As ASD and SZ are neurodevelopmental disorders, understanding the onset of potential changes in metabolite levels and the onset of the changes in microbiome profiles will be insightful and necessary to better understand the link between the brain and the gut during development.

Understanding the diversity and characteristics of gut microbiota is crucial for insights into neurotypical development, disease risk, and treatment efficacy. Our findings combined with data from previous research in animal models and humans collectively highlight the therapeutic potential of targeting gut microbiota in the management of SZ and ASD, offering promising strategies to alleviate both core and comorbid symptoms through microbiome-based interventions in a sex-dependent manner.

## Data Availability

The datasets presented in this study can be found at NCBI under BioProject: PRJNA1274925. Data analysis pipelines, statistics, and figures code can be found at https://github.com/echinodermatamata/frontiers_16_s_microbiome.

## Supplementary material

Supplementary Figure 1 Bacteria identified in female and male Shank3B^+/+^ and Shank3B^+/-^ mice. Percentage bar graph of different bacterial phylum **(A)**, class **(B)**, order **(C)** and genus **(D)** sequenced from the small intestine 3 wild type male mice (Shank3B^+/+^), 3 wild type female mice (Shank3B^+/+^), 3 heterozygote małe mice (Shank3B^+/-^), and 3 heterozygote female mice (Shank3B^+/-^).
