# Supplementary figures and images for: Gut microbiota diversity is altered in a sex-dependent manner in Shank3B heterozygote mice
Source: Front Microbiomes. 2025 Jun 30;4:1628819. doi: 10.3389/frmbi.2025.1628819 (PMC12993676; doi:10.3389/frmbi.2025.1628819)

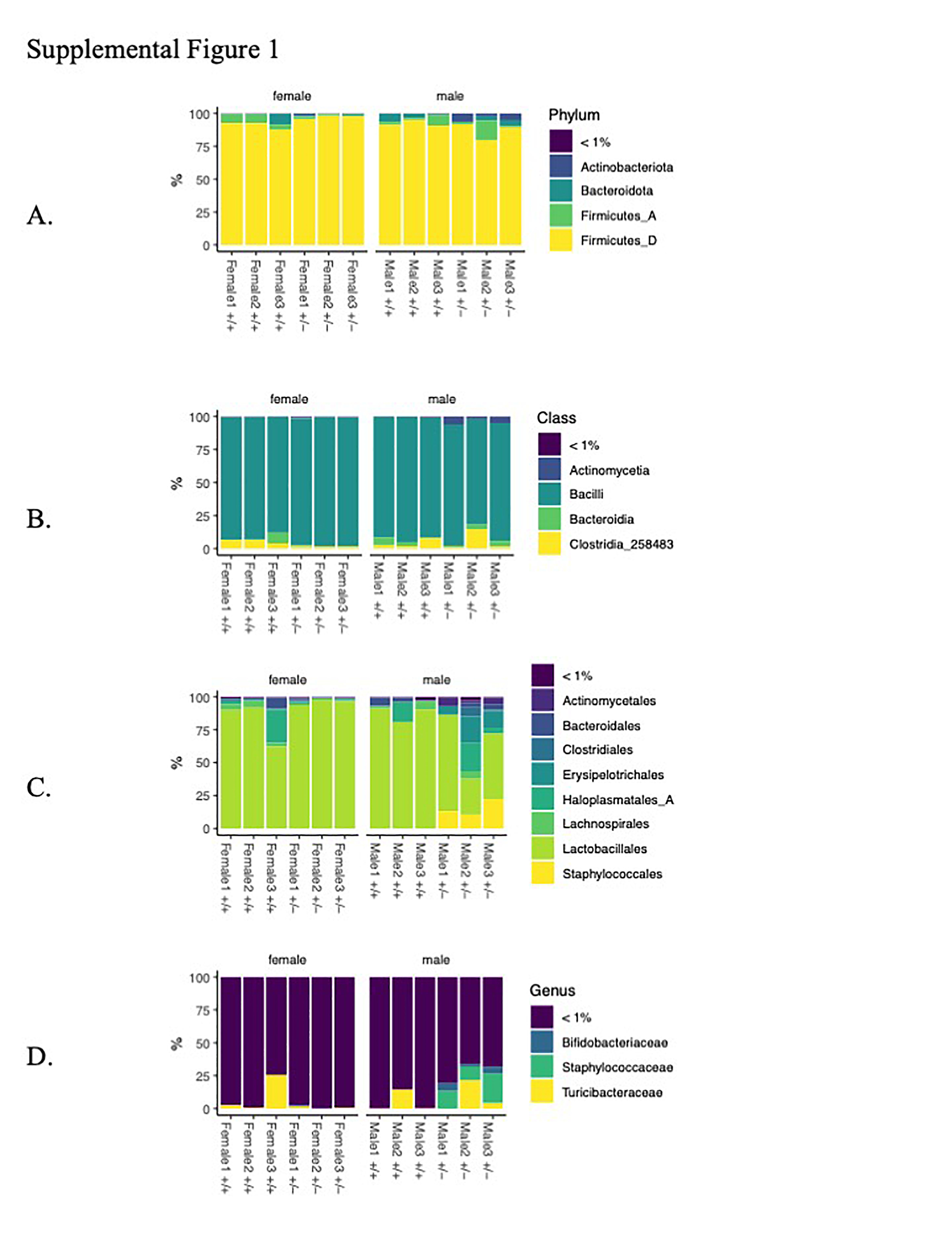

Supplement: Supplementary Figure 1 — Bacteria identified in female and male Shank3B+/+ and Shank3B+/- mice. Percentage bar graph of different bacterial phylum (A), class (B), order (C) and genus (D) sequenced from the small intestine 3 wild type male mice (Shank3B+/+), 3 wild type female mice (Shank3B+/+), 3 heterozygote małe mice (Shank3B+/-), and 3 heterozygote female mice (Shank3B+/-). [file Image1.jpeg]
